# Low rate of dermatology outpatient visits in Asian-Americans: an initial survey study for associated patient-related factors

**DOI:** 10.1186/1471-5945-14-13

**Published:** 2014-08-02

**Authors:** Bharathi Lingala, Shufeng Li, Ashley Wysong, Allison K Truong, David Kim, Anne Lynn S Chang

**Affiliations:** 1 Department of Dermatology, Stanford University, Pavilion C, 2nd Floor, 450 Broadway St, Redwood City 94063, CA, USA

**Keywords:** Dermatology, Skin cancer, Early detection, Acculturation, Asian-Americans, Skin self-examination, Dermatology visits, Prevention, Ethnic skin

## Abstract

**Background:**

Asian-Americans represent the fastest growing minority group in the United States, but are under-represented patients in outpatient dermatology clinics. At the same time, skin cancer rates in individuals of Asian descent are increasing, but skin cancer detection appears to be delayed in Asian-Americans compared to white individuals. Some health-care provider related factors for this phenomenon have been reported in the literature, but the patient-related factors are unclear.

**Methods:**

This exploratory study to identify patient-related factors associated with dermatology visits in Asian-Americans was performed after Institutional Review Board (IRB) approval. An anonymous, online survey utilizing validated items was conducted on adults who self-identified as Asian-American in Northern California. Univariate and multivariate logistic regression for dermatology visits as indicated by responses to the question of “ever having had skin checked by a dermatologist” were performed on survey responses pertaining to demographic information, socioeconomic factors, acculturation, knowledge of melanoma warning signs and SSE belief and practice.

**Results:**

89.7% of individuals who opened the online survey completed the items, with 469 surveys included in the analysis. Only 60% reported ever performing a SSE, and only 48% reported ever having a skin examination by a dermatologist. Multivariate models showed that “ever performing SSE” (p < 0.0001), marital status (p = 0.02), family history of skin cancer (p = 0.03) and generation in the United States (p = 0.02) were significant predictors of the primary outcome of “ever had skin checked by a dermatologist”.

**Conclusions:**

Identification of patient-related factors that associate with dermatology clinic visits in Asian-Americans is important so that this potential gap in dermatologic care can be better addressed through future studies.

## Background

According to the 2012 United States Census Bureau, Asian-Americans currently represent the fastest growing minority group [1]. However, data from the National Ambulatory Medical Care Survey (NAMCS) in 2010 showed that Asian-American represent only 1.07% of ambulatory care visits to dermatologists even though they comprise 3% of the American population. This is potentially concerning as the overall incidence rate of melanoma appears to be rising at 2.4% per year from 1999–2006 in the U. S. in all races [2,3], a trend also seen in worldwide populations where Asians are the majority [4-6]. In the U. S., data from the National Cancer Institute Surveillance, Epidemiology and End Results Program (SEER) database 2007–2011 showed age-adjusted new melanoma cases per 100,000 persons was 8.1 for Asian/Pacific Islanders (52.3 for whites). However, Asians and Pacific Islanders had a higher rate of thicker and distant melanomas compared to whites and Hispanics [2,7-10]. Asians/Pacific Islanders in the U.S. also had lower 5-year survival rates than whites [2].

Factors related to delayed diagnosis could be either patient-related or provider-related. Provider-related factors reported include a lowered index of suspicion among clinicians and different anatomic sites leading to advanced stages at presentation and a poorer prognosis [2]. Patient-related factors are less clear. One hypothesis is that Asian-Americans visit dermatology clinics at a lower rate than other races, as suggested by the NAMCS data. Although there is data on patient characteristics such as personal skin cancer history, skin type, psychosocial factors that associate with clinical skin examinations [11], there is no data in Asian or Asian-American populations. Furthermore, cutaneous examination by a dermatologist is known to impact skin cancer stage at initial presentation [12-17].

This study examines factors that correlate with “ever having skin checked by a dermatologist” in a Northern California Asian-American population. Because of the overwhelmingly outpatient nature of dermatology, having skin checked by a dermatologist almost always occurs in the setting of an outpatient dermatology clinic visit. In addition, we acknowledge that the guidelines for frequency and utility of dermatologists’ cutaneous examinations particularly in asymptomatic people of color continues to be debated due to the lack of high quality evidence for this practice [3,15-18].

## Methods

The study was approved by the Stanford Human Subjects Panel and the need for a written consent was waived as the study was anonymous and voluntary. An online survey study of adults in Northern California who self-identified as Asian-American was performed and included items on demographic information, socioeconomic factors, acculturation, belief in SSE, having heard of melanoma warning signs, SSE practices and ever having skin checked by dermatologist. Survey items were adapted from previously published skin cancer survey instruments [19] and previously published acculturation tools [20-22]. Additional file 1: Figure S1 shows the actual survey instrument, with items used in the study analysis highlighted. Because of the many languages spoken within the Asian population in northern California, the survey was available in English only. To minimize bias toward Asian-Americans with access to dermatologic care, this study was conducted completely online rather than at dermatology clinics. The survey was placed online via the Stanford University Surveyor Web site. The web site address was distributed to leaders of Northern California community groups that were likely to contain a large portion of Asian-Americans for circulation among community group members to identify participants for this study. These group members were all adults. Because of technical limitations, we could not prevent individuals who were under 18 years of age or not Asian-American from accessing the survey. These individuals were excluded prior to statistical analysis (see Results section).

Because of the online format, a conventional response rate could not be calculated. However, we did track the number of participants who completed the survey divided by the number of clicks (hits) from unique Internet Protocol (IP) addresses for this web site regardless of whether any survey items were completed.

Characteristics of the study subjects were summarized using descriptive statistics. Clinically meaningful variables were chosen for analysis and based on review of the medical literature [23-25]. Univariate and multivariate logistic regressions were performed on the primary outcome variable of “ever had skin checked by a dermatologist”.

A final multivariate analyses model on the primary outcome variable of “ever had skin checked by a dermatologist” was generated after checking for multi-collinearity or interactions between any two variables, then applying stepwise selection, plus the variables of interest. Of note, while “generation in the United States” and “self-rated acculturation” did not meet the threshold for collinearity, there was a significant association between these two variables. Hence, only “generation in the United States” was included in the final model. All statistical analyses were conducted using SAS statistical software package (Version 9.1, SAS Institute, Inc., Cary, North Carolina).

## Results

Of the 564 individuals who visited the survey website, 506 (89.7%) completed the survey. To ensure an Asian-American population, individuals who self-identified as only “white” (n = 27) or “other” (n = 10) were excluded from the study. The final study sample included 469 individuals. Table 1 shows the demographic characteristics of this group. The most common self-identified races in the study sample were Chinese (39%), Japanese (18%), Taiwanese (8%), Filipino (7%), Korean (7%), and Vietnamese (6%). Other groups represented (<6% each) include Pacific Islander, Thai, Laotian, Cambodian, African-American, and Caucasian (individuals were allowed to select more than one race or ethnicity to allow for mixed race). Thirty-four percent of individuals were born outside of the United States (first-generation), while 37%, 9%, 6%, and 2% self-identified as second, third, fourth, and fifth generation Americans, respectively. To further assess the degree of cultural identification, respondents were also asked to self-rate their acculturation, with 15% identifying their acculturation as “mostly Asian”, 10% as “very Asian”, 34% as “bicultural”, 19% as “mostly westernized”, and 10% as “very westernized”.

**Table 1:** Characteristics of study participants, with n = 469 responses included unless otherwise indicated

**Demographic and socioeconomic factors**	**Number (%)**
**Age in years, mean (SD)**	37.2 ± 15.9
**Gender**	
Male	147 (31%)
Female	322 (69%)
**Marital status**	
Single	240 (51%)
Married	187 (40%)
Separated, Divorced, Widowed	28 (6%)
Domestic union (living together)	14 (3%)
**Education**	
High School	34 (7%)
College	249 (53%)
Graduate or Professional	180 (38%)
Grade school or no formal education	6 (1%)
**Annual household income** (n = 441, missing data n = 18)	
< $25,000	67 (15%)
$25,000 - $50,000	77 (17%)
$50,001 - $75,000	73 (17%)
$75,001 - $100,000	62 (14%)
> $100,000	162 (37%)
**Has health insurance**	419 (89%)
**Acculturation, n = 410**	
**Generation in the United States**	
First generation (born outside US)	160 (39%)
Second Generation	175 (43%)
Third Generation	41 (10%)
Fourth Generation	26 (6%)
Fifth Generation	8 (2%)
**Self-rated acculturation**, n = 410 (missing response n = 59)	
Very Asian	46 (11%)
Mostly Asian	72 (18%)
Bicultural	159 (39%)
Mostly Westernized	88 (21%)
Very Westernized	45 (11%)
**Additional parameters**	
**Personal history of skin cancer**	8 (1.7%)
**Family history of skin cancer**	19 (4.1%)
**Co-worker or friend with history of skin cancer**	116 (25%)
**Has heard of ABCDEs of melanoma**	79 (16.8%)
**Belief in skin self-examination** (n = 466, missing response n = 3)	
Disagree	82 (18%)
Neutral	97 (21%)
Agree	287 (61%)
**Ever performed skin self-examination (SSE)**	
Never	159 (34%)
1-3 times	144 (31%)
4-6 times	39 (8%)
7-9 times	5 (1%)
10 or more times	94 (20%)
Not sure	28 (6%)
**Ever had skin checked by dermatologist**	
Never	237 (51%)
1-3 times	155 (34%)
4-6 times	38 (8%)
7-9 times	5 (1%)
10 or more times	23 (5%)
Not sure	11 (2%)
**Self-assessed risk for getting skin cancer during lifetime,** n = 465	
Mildly agree	185 (40%)
Mildly disagree	41 (9%)
Neither agree nor disagree	72 (15%)
Strongly agree	144 (31%)
Strongly disagree	23 (5%)
**Skin burns when exposed to strong sunshine without protection,** n = 460	
Not at all	32 (7%)
Somewhat	203 (44%)
Very little	118 (26%)
Yes (red, painful burn)	107 (23%)
**Skin tans when exposed to strong sunshine without protection,** n = 452	
Not tan at all - I just burn	15 (3%)
Tan Slightly	86 (19%)
Tan deeply	140 (31%)
Tan moderately	211 (47%)
**Number of blistering sunburns in lifetime,** n = 468	
0	206 (44%)
1 - 2	168 (36%)
3 - 4	65 (14%)
5 or more	29 (6)

Overall, only 48% of respondents reported ever having their skin checked by a dermatologist. Univariate analyses on the primary outcome of “ever had skin checked by a dermatologist” are presented in Table 2. In multivariate analysis, the only variable significantly associated with “ever having skin checked by dermatologist” was “ever performed a skin self-examination”, (p < 0.0012) (Table 2). Only 60% of respondents had ever performed an SSE. In multivariate analysis, a family history of skin cancer and “ever having skin checked by dermatologist” showed a trend toward significance (p = 0.06).

**Table 2:** Univariate and multivariate analyses on the primary outcome variable of “ever had skin checked by a dermatologist”

**Demographic and socioeconomic factors**	**Univariate OR (95% CI)**	**P**	**Multivariate OR (95% CI)**	**P**
**Age (1 year)**	1.02 (1.01 - 1.04)	**<0.0001**	1.00 (0.97 - 1.03)	0.89
**Gender (Female (ref = Male))**	1.63 (1.09 - 2.43)	**0.02**	1.17 (0.62 - 2.21)	0.64
**Marital status**				
*Single*	Ref	**<0.001**	Ref	0.09
*Married*	2.02 (1.37 - 2.97)		2.52 (1.06 - 5.99)	
*Separated, Divorced, Widowed*	3.90 (1.65 - 9.22)		3.05 (0.65 - 14.3)	
**Education**				
*High School*	Ref	0.83	Ref	0.60
*College*	1.12 (0.55 - 2.31)		1.84 (0.55 - 6.11)	
*Graduate/Professional School*	1.00 (0.48 - 2.10)		1.62 (0.46 - 5.72)	
**Annual household income**				
*< $25,000*	Ref	**0.004**	Ref	0.21
*$25,001-50,000*	1.61 (0.79 - 3.28)		1.83 (0.65 - 5.14)	
*$50,001-75,000*	3.24 (1.59 - 6.61)		3.01 (1.03 - 8.79)	
*$75,001-100,000*	2.98 (1.43 - 6.23)		3.33 (1.10 - 10.07)	
*> $100,000*	2.68 (1.44 - 4.99)		2.19 (0.86 - 5.62)	
**Has health insurance**	0.77 (0.38 - 1.57)	0.47	0.62 (0.21 - 1.84)	0.39
**Acculturation**				
**Generation in the United States**				
*First Generation (born outside US)*	Ref	**<0.0001**	Ref	
*Second Generation*	1.92 (1.23 - 2.99)		1.83 (0.92 - 3.61)	0.40
*Third Generation*	3.68 (1.77 - 7.65)		1.52 (0.45 - 5.15)	
*Fourth or more Generation*	2.99 (1.37 - 6.52)		1.63 (0.52 - 5.04)	
**Self-rated acculturation**				
*Very Asian*	Ref	**0.001**	Ref	0.44
*Mostly Asian*	2.52 (1.12 - 5.67)		0.94 (0.34 - 2.56)	
*Bicultural*	2.15 (1.03 - 4.47)		1.09 (0.37 - 3.22)	
*Mostly Westernized*	4.82 (2.17 - 10.67)		1.81 (0.59 - 5.58)	
*Very Westernized*	3.76 (1.52 - 9.34)		1.83 (0.48 - 6.92)	
**Additional parameters**				
**Personal history of skin cancer**	7.71 (0.94 - 63.11)	0.06	NR*	
**Family history of skin cancer**	3.92 (1.27 - 12.05)	**0.02**	5.67 (0.94 - 34.08)	0.06
**Co-worker or friend with history of skin cancer**	2.37 (1.52 - 3.69)	**0.001**	1.05 (0.54 - 2.05)	0.89
**Has heard of ABCDEs of melanoma**	1.78 (1.08 - 2.94)	**0.03**	1.20 (0.57 - 2.53)	0.64
**Belief in SSE**				
*Disagree*	Ref	**<0.0001**	Ref	0.23
*Agree*	2.44 (1.45 - 4.12)		1.29 (0.50 - 3.32)	
*Neutral*	1.05 (0.56 - 1.97)		1.98 (0.84 - 4.67)	
**Ever performed SSE**	4.28 (2.79 - 6.59)	**<0.0001**	2.99 (1.54 - 5.80)	**0.0012**
**Self-assessed risk for getting skin cancer during lifetime,** n = 465				
Strongly disagree	Ref	0.0099	Ref	0.13
Mildly agree	1.34 (0.54 - 3.35)		1.18 (0.28 - 5.00)	
Mildly disagree	1.58 (0.54 - 4.61)		1.72 (0.33 - 8.98)	
Neither agree nor disagree	1.17 (0.43 - 3.15)		0.60 (0.13 - 2.76)	
Strongly agree	2.69 (1.06 - 6.82)		1.99 (0.45 - 8.73)	
**Skin burns when exposed to strong sunshine without protection**				
Not at all	Ref	0.54	Ref	0.05
Somewhat	1.46 (0.67 - 3.17)		1.37 (0.43 - 4.40)	
Very little	1.70 (0.76 - 3.81)		2.53 (0.76 - 8.46)	
Yes (red, painful burn)	1.52 (0.67 - 3.45)		0.77 (0.22 - 2.74)	
**Skin tans when exposed to strong sunshine without protection**				
Not tan at all - I just burn	Ref	0.20	Ref	0.13
Tan Slightly	1.61 (0.51 - 5.12)		0.37 (0.06 - 2.25)	
Tan deeply	2.45 (0.80 - 7.55)		0.85 (0.15 - 4.84)	
Tan moderately	1.76 (0.58 - 5.34)		0.57 (0.10 - 3.15)	
**Number of blistering sunburns in lifetime**				
0	Ref	0.78	Ref	0.73
1 - 2	1.13 (0.75 - 1.71)		1.28 (0.67 - 2.43)	
3 - 4	1.30 (0.74 - 2.28)		1.32 (0.58 - 3.01)	
5 or more	1.28 (0.57 - 2.85)		1.92 (0.49 - 7.49)	

*Not included in multivariate analysis due to small number of skin cancers (n = 8).

Multivariate analysis was based on n = 324 unless otherwise indicated.

Bolded numbers indicate significance at a level P<0.05.

To explore predictors of “having skin checked by a dermatologist”, multiple multivariate analyses varying the variables were performed, after accounting for multi-collinearity or interaction (results shown in Table 3). Significant predictors of “having skin checked by a dermatologist” included Ever Performed SSE (p < 0.0001), marital status (p = 0.02), generation in the United States (p = 0.03), and family history of skin cancer (p = 0.03).

**Table 3:** Final multivariate analyses model on the primary outcome variable of “ever had skin checked by a dermatologist”

**Characteristic**	**Multivariate OR (95% CI)**	**P**
**Education**		0.24
*High School*	Ref	
*College*	2.19 (0.80 - 6.00)	
*Graduate/Professional School*	1.64 (0.57 - 4.74)	
**Annual household income**		0.07
*< $25,000*	Ref	
*$25,001-50,000*	1.93 (0.79 - 4.72)	
*$50,001-75,000*	**3.19 (1.24 - 8.23)**	
*$75,001-100,000*	**3.70 (1.41 - 9.71)**	
*> $100,000*	**2.60 (1.13 - 6.02)**	
**Has health insurance**	0.55 (0.22 - 1.41)	0.22
**Ever performed SSE**	**3.32 (1.95 - 5.65)**	**<0.0001**
**Marital status**		**0.02**
*Single*	Ref	
*Married*	**2.04 (1.13 - 3.70)**	
*Separated, Divorced, Widowed*	**3.56 (1.01 - 12.61)**	
**Family history of skin cancer**	**6.50 (1.15 - 36.69)**	**0.03**
**Generation in the United States**		**0.02**
*First Generation (born outside US)*	Ref	
*Second Generation*	**2.11 (1.20 - 3.72)**	
*Third Generation*	2.21 (0.85 - 5.80)	
*Fourth or more Generation*	**3.14 (1.17 - 8.42)**	

This model was selected after checking for multi-collinearity or interactions between any two variables, then applying stepwise selection, plus the variables of interest. Analysis based on n = 349 responses.

Bolded numbers indicate significance at a level P<0.05.

## Discussion

The rates of “ever had skin checked by dermatologist” in our Asian-American study population are difficult to directly compare with other minority groups. Perhaps the most similar study in the literature was an online survey of Hispanics living in the United States, which showed only 9.2% had a total cutaneous examination [10]. As the overall purpose of our study was to examine patient-related factors associated with dermatologic clinic visits, our current survey did not ask if a patient had a total cutaneous examination, only whether they ever had skin checked by dermatologist. This would likely include skin checks for benign skin disease as well as skin cancer surveillance, hence, accounting for our higher percentage of individuals “ever having skin checked by dermatologist” compared to total cutaneous examinations reported in other studies. It is also difficult to directly compare our study with existing studies in other minority groups due to differences in geographic locales and survey methods.

The study participants were similar to the local Asian-American community with respect to household income, education and health insurance status. For instance, the median household income for single race Asian households in Santa Clara County, the location of our institution, was $68,780 (by the U.S. Census, 2009); for our study, 50% of participants reported incomes above $75,000. For education level in single race Asians in Santa County Clara County, 50% have a bachelors or some college degree; this is 53% in our study. For health insurance in single race Asians in Santa Clara County, 17.2% did not have health insurance; 11% in our survey did not. Hence, this study is likely generalizable to the local Asian-American community.

This study was conducted only in English (due to the large number of languages spoken by Asian-Americans in Northern California) and is therefore biased in favor of those proficient in English. In addition, this study was performed online, hence accessible only to those with computer literacy and Internet access. Finally, the Asian-American community is very heterogeneous and therefore, this study does not necessarily represent the demographic and clinical factors and practices of each subgroup.

Finally, SSE and cutaneous examinations by dermatologists have been significantly linked in previous studies that did not primarily focus on Asian-Americans [26]. Nevertheless, the ideal frequency of SSE and cutaneous examinations in asymptomatic persons of any race has not been established, in part due to the lack of high quality studies to support these measures [3,15-18]. Nevertheless, a recent expert opinion article by Agbai *et al.* in persons of color did suggest monthly self-skin examinations, though no recommendations were made for cutaneous examinations by dermatologists [3].

## Conclusion

Future research is needed to determine the optimal frequency of skin examinations in Asian-Americans and whether increased dermatology clinic visits lead to earlier detection of skin cancers in Asian-Americans.

## Competing interests

The authors have no competing interests to declare.

## Authors’ contributions

BL contributed to conception, design, statistical analysis and data interpretation. AW and AC contributed to the conception, design, data acquisition, statistical analysis, data interpretation, manuscript draft and revision. SL contributed to design, statistical analysis, data interpretation, manuscript draft and revision. AT contributed to data interpretation and manuscript draft and revision. DK contributed to data acquisition and manuscript draft. All authors read and approved the final manuscript.

## Authors’ information

Anne Lynn Chang is an Assistant Professor of Dermatology at Stanford University School of Medicine. She is Director of the Stanford Dermatology Adult Dermatological Clinical Trials, the Advanced Basal Cell Carcinoma Clinic and a member of the Stanford Cancer Institute.

Bharathi Lingala and Shufeng Li are co-first authors.

## Pre-publication history

The pre-publication history for this paper can be accessed here:

http://www.biomedcentral.com/1471-5945/14/13/prepub

## Supplementary Material

Additional file 1: Figure S1 Survey instrument with items included in the analysis highlighted in yellow. Click here for file
