# Supplementary material for: Low rate of dermatology outpatient visits in Asian-Americans: an initial survey study for associated patient-related factors
Source: BMC Dermatol. 2014 Aug 2;14:13. doi: 10.1186/1471-5945-14-13 (PMC4130701; doi:10.1186/1471-5945-14-13)
Supplement: Additional file 1: Figure S1 — Survey instrument with items included in the analysis highlighted in yellow. [file 1471-5945-14-13-S1.pdf]

Skin Cancer Prevention and Detection Among Asians Living in Northern California - Anonymous Survey

* = Required

1. I have heard about the ABCDE rule to look for melanoma skin cancer.
- No
- Yes
- Not Sure

2. I need to check my body for abnormal moles at least once a year.
- Strongly disagree
- Mildly disagree
- Neither agree nor disagree
- Mildly agree
- Strongly agree

3. I need to check my palms and soles of my feet for abnormal moles at least every year.
- Strongly disagree
- Mildly disagree
- Neither agree nor disagree
- Mildly agree
- Strongly agree

4. The rate of new skin cancers in Caucasian(white) individuals in the United states is decreasing.
- Strongly disagree
- Mildly disagree
- Neither agree nor disagree
- Mildly agree
- Strongly agree

5. The rate of new skin cancers in Asians (such as Japan or Singapore) is decreasing.

- Strongly disagree
- Mildly disagree
- Neither agree nor disagree
- Mildly agree
- Strongly agree

6. All skin cancers are dark colored (brown or black).

- True
- False
- Don't know

7. I am at risk for getting skin cancer during my lifetime.

- Strongly disagree
- Mildly disagree
- Neither agree nor disagree
- Mildly agree
- Strongly agree

8. Tanned skin is attractive.

- Strongly disagree
- Mildly disagree
- Niether agree nor disagree
- Mildly agree
- Strongly agree

9. I can get addicted to using a tanning bed.

- Strongly disagree
- Mildly disagree
- Neither agree nor disagree
- Mildly agree
- Strongly agree

10. Ultraviolet light from a tanning bed causes cancer.

- Strongly disagree
- Mildly disagree
- Neither agree nor disagree
- Mildly agree
- Strongly agree

11. In the past, I have been referred to a tanning bed/salon by my manicurist, hair stylist, facialist (esthetician) or massage therapist.

- No
- Yes

12. I know a friend, relative, co-worker or acquaintance who uses a tanning bed/salon.

- No
- Yes

13. On average, how many hours do you spend outdoors in sunlight during a WEEKEND day?

- Less than 1 hour
- 1 to <2 hours
- 2 to <4 hours
- 4 to <6 hours
- 6 or more hours

14. On average how many hours do you spend outdoors in the sunlight during a WEEKDAY?

- Less than 1 hour
- 1 to <2 hours
- 2 to <4 hours
- 4 to <6 hours
- 6 or more hours

15. How many years have you worked at an occupation that required more than 3 hours of outdoor time in the sunlight per day?

- 0 (Never)
- Less than 1 year
- 1 to <2 years
- 2 to <5 years
- 5 to <10 years
- 10 or more years

16. How many years have you participated in a hobby, sport or activity that required more than 3 hours per day outdoors in sunlight?

- 0 (Never)
- Less than 1 year
- 1 to <2 years
- 2 to <5 years
- 5 to <10 years
- 10 or more years

17. How many times have you used a tanning bed in your life?

- 0 (Never)
- 1-3 times
- 4-6 times
- 7-10 times
- 11 or more times

18. How many times in your life have you self-checked your skin for abnormal moles?

- 0 (Never)
- 1-3 times
- 4-6 times
- 7-9 times
- 10 or more times
- Not sure

19. How many times have you had your skin checked by a dermatologist?

- ○ 0 (Never)
- ○ 1-3 times
- ○ 4-6 times
- ○ 7-9 times
- ○ 10 or more times
- ○ Not sure

20. How many times in your life have you received a facial at a salon?

- ○ 0 (Never)
- ○ 1 - 3 times
- ○ 4 - 6 times
- ○ 7 - 10 times
- ○ 11 or more times

21. How many times in your life have you seen a massage therapist?

- ○ 0 (Never)
- ○ 1 - 3 times
- ○ 4 - 6 times
- ○ 7 - 10 times
- ○ 11 or more times

22. How many times have you had a manicure or pedicure at a salon?

- ○ 0 (Never)
- ○ 1 - 3 times
- ○ 4 - 6 times
- ○ 7 - 10 times
- ○ 11 or more times

23. Where do you get MOST of your information about skin care or health? Check all that apply.

- ☐ Internet

- [ ] Beauty salon, Barber, or Spa (hairstylist, manicurist or facialist)
- [ ] Doctors or Nurses
- [ ] TV, Magazines, Newspaper, or Radio
- [ ] Family
- [ ] School
- [ ] Work
- [ ] Other

24. How often do you seek shade from the sun when outdoors?

- ○ Never
- ○ Rarely
- ○ Sometimes
- ○ Most of the time
- ○ All of the time

25. How often do you wear a hat or long sleeve clothing when you go out in the sun?

- ○ Never
- ○ Rarely
- ○ Sometimes
- ○ Most of the time
- ○ All of the time

26. How often do you wear sunscreen when you know you will be in the sun?

- ○ Never
- ○ Rarely
- ○ Sometimes
- ○ Most of the time
- ○ All of the time

27. How often do you put sunscreen or hats on your children (<18 years old)?

- ○ Does not apply - I have no children
- ○ Never

- A few times a year
- A few times a month
- A few times a week
- Daily

28. What year were you born (please give a 4 digit answer)?*

29. Are you male or female?*
- Male
- Female

30. Ethnicity (if you are mixed races, check all that apply):*
☐ African-American ☐ Alaskan Native ☐ American Indian ☐ Burmese ☐ Cambodian ☐ Caucasian (white ☐ Chinese ☐ Filipino ☐ Latino/Hispanic ☐ Indian ☐ Japanese ☐ Korean ☐ Laotian ☐ Malaysian ☐ Pacific Islander ☐ Pakistani ☐ Taiwanese ☐ Thai ☐ Vietnamese ☐ Other

31. What is your marital status?
- Single (never married)
- Divorced
- Widowed
- Separated
- Married
- Domestic Union (living together)

32. My highest level of education is:
- Did not attend formal schooling
- Grade school (grades 1-8)
- High school (grades 9-12)
- college
- graduate or professional school

33. How would you describe your combined household income per year on

average?

- Less than $25,000
- $25,001-50,000
- $50,001-75,000
- $75,001-$100,000
- Greater than $100,000

34. When exposed to the strong sun in summer without protection would your skin burn?

- Not at all
- Very little
- Somewhat
- Yes (red, painful burn)
- Don't know

35. When exposed to strong sun in summer without protection your skin would:

- Not tan at all - I just burn
- Tan Slightly
- Tan moderately
- Tan deeply
- Don't know

36. How many blistering sunburns have you had in your lifetime?

- 0
- 1-2
- 3-4
- 5 or more

37. Have you ever had skin cancer?

- No
- Yes
- Don't know

38. I have had the following types of skin cancer:

- This question does not apply to me-- I have never had skin cancer
- Melanoma
- Basal cell
- Squamous cell
- Other type
- Don't know the type

39. Has anyone in your family had skin cancer?

- No
- Yes
- Don't know

40. Have any of your friends or co-workers had skin cancer?

- No
- Yes
- Don't know

41. Do you have health insurance?

- No
- Yes
- Don't know

42. Is it hard for you to get an appointment with a dermatologist?

- No
- Yes
- Don't know

43. If you are Asian-American, what generation are you? If you are not Asian-American, please skip this question.

- First generation (born in Asian or country other than U.S.)
- Second generation (I was born in U.S.; either parent was born in Asia or country other than U.S.)
- Third generation (I was born in the U.S.; both parents were born in U.S., and

all grandparents born in Asia or country other than U.S.)

- Fourth generation (I was born in the U.S.), both parents were born in the U.S., and at least one grandparent was born in Asia or country other than U.S. and one granparent born in U.S.
- Fifth generation (I was born in the U.S., both parents were born in U.S., all grandparents also born in U.S.)
- Don't know what generation best fits since I lack some information

44. If you are Asian-American, where were you raised? If you are not Asian-American, skip this question.

- In Asia only
- Mostly in Asia, some in U.S.
- Equally in Asia and U.S.
- Mostly in U.S., some in Asia
- In U.S. only

45. What contact have you had with Asia? If you are not Asian-American, skip this question.

- Lived one year or more in Asia
- Lived for less than one year in Asia
- Occasional visits to Asia
- Communications (letters, phone calls, internet, etc) with people in Asia
- No exposure or communications with people in Asia

46. How would you rate yourself? If you are not Asian-American, please skip this question.

- Very Asian (I consider myself basically an Asian person, even though I live and work in the U.S.)
- Mostly Asian (I consider myself Asian-American, though deep down I always know I am Asian)
- Bicultural (I have both Asian and American characteristics, I view myself as a blend of both Asian and American)
- Mostly Westernized (I consider myself Asian-American, although deep down I view myself as an American first)
- Very Westernized (I consider myself basically American, even though I have an Asian background and characteristics)

This Survey is NOT yet functional

©2010 Stanford School of Medicine | Terms of Use | Powered by
